# Detection of temporal, spatial and spatiotemporal clustering of malaria incidence in northwest Ethiopia, 2012–2020

**DOI:** 10.1038/s41598-022-07713-3

**Published:** 2022-03-07

**Authors:** Teshager Zerihun Nigussie, Temesgen T. Zewotir, Essey Kebede Muluneh

**Affiliations:** 1 Department of Statistics, College of Science, Bahir Dar University, Bahir Dar, Ethiopia; 2 School of Mathematics, Statistics and Computer Science, College of Agriculture Engineering and Science, University of KwaZulu-Natal, Durban, South Africa; 3 Department of Public Health, College of Medicine and Health Sciences, Bahir Dar University, Bahir Dar, Ethiopia

**Keywords:** Diseases, Risk factors

## Abstract

Malaria is one of Ethiopia's most targeted communicable diseases for elimination. Malaria transmission varies significantly across space and time; and Ethiopia had space–time disparity in its transmission intensities. Considering heterogeneity and transmission intensity at the district level could play a crucial role in malaria prevention and elimination. This study aimed to explore temporal, spatial, and spatiotemporal clusters of malaria incidence in northwest Ethiopia. The analysis is based on monthly malaria surveillance data of districts and collected from the Amhara public health institute. The Kulldorff's retrospective space–time scan statistics using a discrete Poisson model were used to detect temporal, spatial, and space–time clusters of malaria incidence with and without adjusting the altitude + LLIN arm. Monthly malaria incidence had seasonal variations, and higher seasonal indices occurred in October and November. The temporal cluster occurred in the higher transmission season between September and December annually. The higher malaria incidence risk occurred between July 2012 and December 2013 (LLR = 414,013.41, RR = 2.54, P < 0.05). The purely spatial clustering result revealed that the most likely cluster occurred in the north and northwest parts of the region while secondary clusters varied in years. The space–time clusters were detected with and without considering altitude + LLIN arm. The most likely space–time cluster was concentrated in northwestern and western parts of the region with a high-risk period between July 2012 and December 2013 (LLR = 880,088.3, RR = 5.5, P < 0.001). We found eight significant space–time clusters using the altitude + LLIN arm. The most likely space–time cluster occurred in the western and northwestern parts of the region in July 2012–December 2013 (LLR = 886,097.7, RR = 5.55, P < 0.05). However, secondary clusters were located in eastern, northwestern, western parts of regions, which had different cases and relative risks in each cluster. Malaria transmission had temporal, spatial, and space–time variation in the region at the district level. Hence, considering these variations and factors contributing to malaria stratification would play an indispensable role in preventing and controlling practices that ultimately leads to malaria eliminations.

## Introduction

Malaria is one of Ethiopia's most targeted communicable diseases for elimination. Three-quarters of the country's land is malarious, and about two-thirds of the population lives in malaria-prone areas^1,2^. Ethiopia has a wide range of malaria transmission intensities ranging from low seasonal to high perennial transmissions. Malaria transmission is seasonal and unstable in Ethiopia^3^. A larger number of cases occurred mostly from September to December, following the main rainy season in July and August. The second peak cases occurred in May and June, following a short rainy season in April and May^4^. Districts, third level administrative level, were classified into four malaria transmission strata: malaria-free, low, moderate, and high transmission^5^. The transmission showed significant space–time variations, and regions also had significantly varied malaria transmission intensities in Ethiopia^1,3,6,7^.

Malaria disease is a major health facility concern in the Amhara region, located in the northwest of Ethiopia, severely affecting the society in the main harvesting season. In 2012, malaria prevalence was 60 per 1000 people and accounted the 19% of the national malaria burden^1,8,9^. The malaria incidence had spatial, seasonal, and space–time heterogeneity across districts with unstable transmission^1,10^. Malaria transmission is heterogeneous across areas and time due to complex interactions among parasites, vectors, and hosts. The altitude, weather conditions, coverage of long-lasting insecticide nets, indoor residual spraying, and other societal and government interventions contribute to variations of malaria transmission^6,11^.

Effective malaria intervention requires a good understanding of transmission dynamics in space and time to assist malaria control programs^12^. Considering heterogeneity and transmission intensity of malaria could play a critical role in improving malaria intervention strategy and control measures. In Ethiopia, fewer studies were conducted to detect temporal, spatial, and spatiotemporal clustering of malaria incidence of areas with lower and higher malaria transmission. Taddese et al.^10^ found that the southwest parts of the region had higher malaria risks, and all districts in the north Gondar zone had higher risk between 2015/7/1 to 2016/12/31. A study in southwest Ethiopia revealed that the most likely spatial cluster occurred in the south Gondar zone and covered five districts. The most likely spatiotemporal cluster also occurred at the most likely spatial cluster between January 2003 and December 2012^1^. In southern-central Ethiopia, spatial and spatiotemporal clustering of malaria was detected using LLIN + IRS arms. The risk of malaria was varied across kebele, within kebele, and among households^13^.

The spatial and spatiotemporal heterogeneity of malaria transmissions were investigated at household and district levels^3,8,10,13–19^. However, spatial and spatiotemporal clustering of areas having lower and higher malaria transmission was not fully explored. Few studies used long-lasting insecticide nets and indoor residual spraying to adjust the space–time clustering of areas in southern-central Ethiopia^13^. In this study, spatiotemporal clustering of districts was adjusted to the average altitude of districts (< 2000 m, 2000 – 2500 m, > 2500 m) and coverage of at least one long-lasting insecticide net at the household level in northwest Ethiopia. Analyzing and assessing the spatiotemporal patterns and clusters of malaria incidents in this study is played an essential role in malaria control and elimination. This study aimed to explore the temporal, spatial, and spatiotemporal clusters of malaria incidence in the districts of the Amhara region using Kulldorff's scan statistics.

## Methods

### Study area

**Figure 1:**
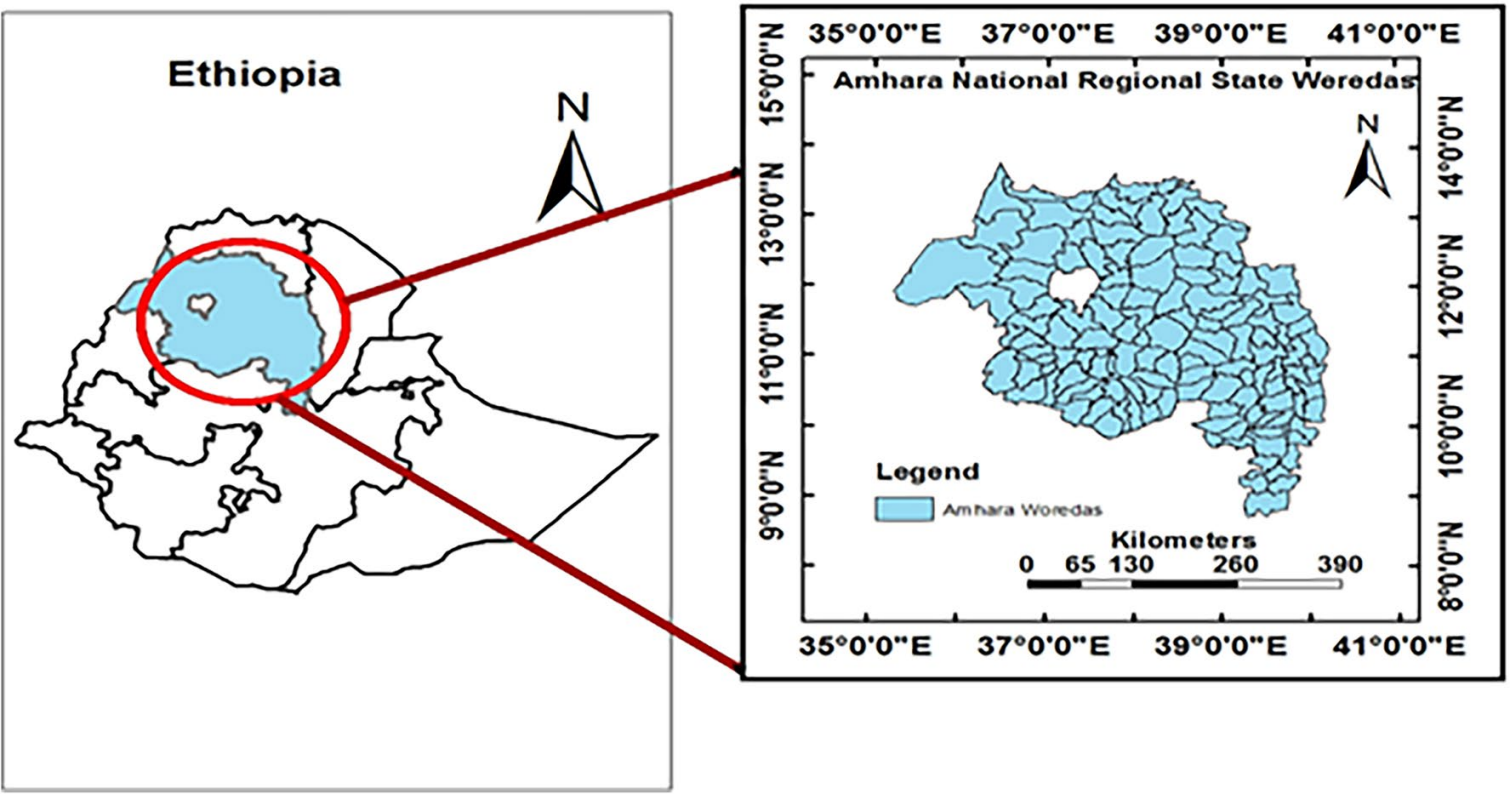
Study area map, districts in the Amhara National Regional State, Ethiopia in 2012 (ArcGIS, v10.3; https://www.esri.com/en-us/arcgis/products/arcgis-desktop/resources).

This study was conducted in the Amhara regional state, Ethiopia. The region is located in the northwest of Ethiopia between 9° 20ʹ and 14° 20ʹ North latitude and 36° 20ʹ and 40° 20ʹ East longitude. The altitude of the study region ranges from 506 m at the bottom of the Blue Nile gorge to 4533 m at Ras-Dajen mount^20^. The study region encompassed 152 districts in early 2012, including the municipality, cities, and rural administrations districts (Fig. 1).

### Data

In this study, the monthly malaria incidence of 152 districts between July 2012 and June 2020 was used to explore spatiotemporal clusters. Monthly malaria cases of districts were obtained by adding weekly malaria surveillance data. The weekly malaria surveillance data of districts, town administrations, general and specialized hospitals in the Amhara region were obtained from the Amhara Public Health Institute (APHI). The weekly malaria data were reported using WHO epidemiology-week (EPI-week), which is starting on Sunday. The fiscal year of Ethiopia is started on 8th July and ends on 7th July. The EPI-weeks of APHI disease surveillance reporting year ran from EPI-week 28 falling in July to EPI-week 27 at the first week of July. In this study, monthly total malaria case was obtained by adding the weekly surveillance data with the considerations of an EPI-week had at least four days that fall in a given month.

The polygon shapefile of the study region was obtained from a central statistical agency. The polygon shapefile was converted to point coordinate (latitude and longitude) using ArcGIS10.3^21^. The estimated population size of districts was collected from the central statistical agency and Amhara National Regional State Planning Commission and combined with the district polygon shapefiles. The population data were used to compute the expected number of malaria incidence to identify spatial, space–time clusters^22^.

### Statistical methods

The data were analyzed using Global Moran’s statistics, seasonal decomposition, and Kulldorff’s retrospective space–time scan statistics.

### Global Moran's *I* value

The Global Moran's *I* spatial autocorrelation was used to measure and test the overall spatial clustering tendency of annual malaria incidence in northwest Ethiopia*.* The Moran's *I* statistic takes a value between − 1 and 1. If close to 1 indicates positive autocorrelation, and districts are clustered based on their malaria transmission intensity. Moran’s I statistic is less than zero indicates negative autocorrelation, and the closer it is to − 1, the more dispersed distribution^23^. Moran’s *I* = 0 suggests that the disease is randomly distributed across the districts of the study region. The Moran’s I statistic is evaluated by the Monte-Carlo test using Z statistic and P-value. If P-value is less than 5% level of significance indicates the presence of significant spatial autocorrelation or scattering of cases across space. The Global Moran's *I* statistic is computed as follows:

$${\text{Moran}}^{\prime}{\text{s}}\,\,I = \frac{{n\mathop \sum \nolimits_{i = 1}^{d} \mathop \sum \nolimits_{j = 1}^{d} w_{ij} (x_{i} - \overline{x})\left( {x_{j} - \overline{x}} \right)}}{{\left( {\mathop \sum \nolimits_{i = 1}^{d} \mathop \sum \nolimits_{j = 1}^{d} w_{ij} } \right)\mathop \sum \nolimits_{i = 1}^{d} \left( {x_{i} - \overline{x}} \right)}}, E\left( I \right) = \frac{1}{n - 1}.\,\,\, Z = \frac{I - E\left( I \right)}{{\sqrt {Var\left( I \right)} }}$$

where d is the number of districts; $x_{i}$ and $x_{j}$ are the incidence rates in districts $i$ and $j$; $\overline{x}$ is the mean incidence rate in the entire region; and $w_{ij}$ is the spatial weight between districts $i$ and $j$. We generated a binary spatial weight matrix using the Rook contiguity rule, assigned 1 when districts are neighbors and 0 otherwise^23^.

### Seasonal decomposition

The time series decomposition describes the trend and seasonal factors in the time series of monthly malaria incidence in northwest Ethiopia. We conducted a seasonal decomposition analysis to assess seasonal patterns, and the seasonal index of malaria has also been estimated in the study region^24^. A seasonal index is used to examine the seasonal pattern of malaria incidences, and a value approach to 1.0 revealed the absence of obvious seasonal patterns in the data. The seasonal index is the average number of cases for a given month divided by the average monthly cases during the study periods^25^.

### Spatiotemporal cluster analysis

The temporal, spatial, and space–time clusters of malaria incidence were identified using Kulldorff’s retrospective space–time scan statistics^26^. Monthly malaria cases of districts are counts; hence a discrete Poisson model was employed to scan spatiotemporal clusters of malaria incidences^1^. All possible space–time clusters were detected using a dynamic circular window scanned in temporal and geographic dimensions. The radius of the circular geographic base varies with the area's population range, while the height fluctuates with the set period^27^.

The scanning window's relative risk (RR) is computed by taking the ratio of the observed and expected number of cases within and outside of the given scanning windows. The P-value of clusters was calculated through 9999 Monte Carlo simulations, and clusters with less than a 5% level of significance are considered potential high-risk clusters^28^. The window having the largest log-likelihood ratio (LLR) is defined as the most likely cluster. The remaining windows with a statistically significant LLR are called secondary clusters and ranked according to their LLR value^29^. The LLR of a given cluster is calculated using:

$$LLR = {\text{log}}\left( {\frac{n}{E\left( n \right)}} \right)^{n} \left( {\frac{N - n}{{N - E\left( n \right)}}} \right)^{N - n} I^{\prime}$$

where N is the total number of cases; n is the observed number of cases in the scanning window; $E\left( n \right)$ and $N - E\left( n \right)$ are expected number of cases within and outside the scanning window under the null hypothesis ($H_{0} :$ The spatiotemporal clustering of the study areas is caused by random factors.), respectively; and $I$ is an indicator function. It takes 1 when the window has more cases than expected under the null hypothesis; otherwise, 0.

The space–time scan results are sensitive to selecting the spatial and temporal scanning window's maximum radius and the maximum length of the population under study^30^. The Gini coefficient is used to establish the ideal maximum reported cluster sizes, which improves spatial scan statistics by providing a criterion for selecting the collection of non-overlapping clusters to report. The Gini coefficient is a simple and easy approach to assess the degree of heterogeneity in a collection of spatial clusters, which is useful for determining how well the cluster collection reveals the underlying cluster patterns underneath^31^. The spatial clusters of annual malaria incidence were identified using optimal parameters of the cluster sizes from 1 to 25% of the total population at risk by an increment of 1%. The radius was considered optimal for analysis if there were fewer overlaps between areas defined by the radius. The biggest cluster covered less than 15% of the districts included in the study^32,33^.

The space–time clusters can be detected by scanning using up to 50% of the population at risk^30^. This study used a spatial and temporal scanning window size of 30% of the total population at risk and the total study period to detect smaller to larger clusters. The time frame of the space–time scan analysis was set at one month, and time trends and observed clusters changed in the entire study period.

Malaria transmissions were heterogeneously varied across geography due to the complex interaction of host, parasite, vectors, and environmental conditions. Further, usage of long-lasting insecticide nets (LLIN) and indoor residual spraying play a vital role in malaria prevention and control^34^. Detecting space–time clusters of areas with higher malaria transmission risks adjusting to the altitude and annual coverage of at least one LLIN at the household level are clusters that could not be explained by the altitudinal variation and coverage of LLIN at the household level and ultimately played an indispensable role in malaria control and preventive measures. Hence, spatiotemporal clusters adjusted to the average altitude and LLINS were also determined using Kulldorff’s retrospective space–time scan statistics using a discrete Poisson model.

The SaTScan™ software (v 10.0, Kulldorff and Information Management Service, Inc.) was used for spatial, temporal, and spatiotemporal clustering analysis. We used ArcGIS (v10.3 ESRI Inc, CA, USA) to visualize malaria incidence relative risk (RR) in high-risk cluster areas and the presence of significant global spatial autocorrelations. The R 4.0 was also used to decompose and estimate the seasonal indecies of monthly malaria cases of the study region between 2012 and 2020.

### Ethical clearance

This research protocol was approved by the College of Science Ethical Review Board, Bahir Dar University, with a letter referenced as PGRCSVD/137/201. The ethical review board waived participant informed consent since the study was conducted using district-level monthly aggregated malaria data.

### Institutional review board statement

The study was conducted according to the guidelines of the Declaration of Helsinki, and approved by the Ethics Committee of College of Science, Bahir Dar University (protocol code: PGRCSVD/137/201, and date of approval: July 9, 2020).

## Results

In 2012–2020, more than 4.5 million reported malaria cases occurred in northwest Ethiopia. The study area included different ecological zones ranging from lower to higher malaria transmission suitable areas where most are prone to malaria. This study aimed to detect areas where and when the malaria incidence occurred at a higher risk than expected by chance. The space–time scan detection of clusters was considered with and without adjusting to the altitude (< 2000, 2000–2500, > 2500 m) and coverage of LLIN arms. The spatial autocorrelation was estimated using Global Moran's *I* statistic, and the result for each year is given in Table 1. The result revealed that annual malaria incidences had positive spatial autocorrelation between 2012 and 2020, ranging from 0.317 to 0. 513 (all P < 0.05).

**Table 1:** Global Moran's I autocorrelation value for annual malaria cases in northwest Ethiopia, 2012–2020.

Year	Moran’s I	Variance	Z-score	P
2012	0.457	0.0023	9.599	0.000
2013	0.513	0.0024	10.658	0.000
2014	0.480	0.0022	10.290	0.000
2015	0.439	0.0023	9.395	0.000
2016	0.432	0.0022	9.134	0.000
2017	0.449	0.0021	9.958	0.000
2018	0.505	0.0021	11.052	0.000
2019	0.413	0.0023	8.779	0.000
2020	0.317	0.0020	7.164	0.000

The optimal temporal scanning window was the time window with the length covering 10–50% of the total study period by an increment of 5%. Temporal scanning windows ranging from 25 to 50% of the study period provided identical temporal clusters. We determined the maximum temporal cluster sizes by setting the temporal scanning window size at 30% of the total study period. The maximum spatial cluster sizes of the purely spatial scan statistics were determined using the Gini coefficient, and optimal scanning window size was selected using SaTScan. The optimal maximum spatiotemporal clusters were determined at 30% of the total study population, and the spatial and temporal scanning radius was set as 30%.

### Temporal cluster analysis

**Figure 2:**
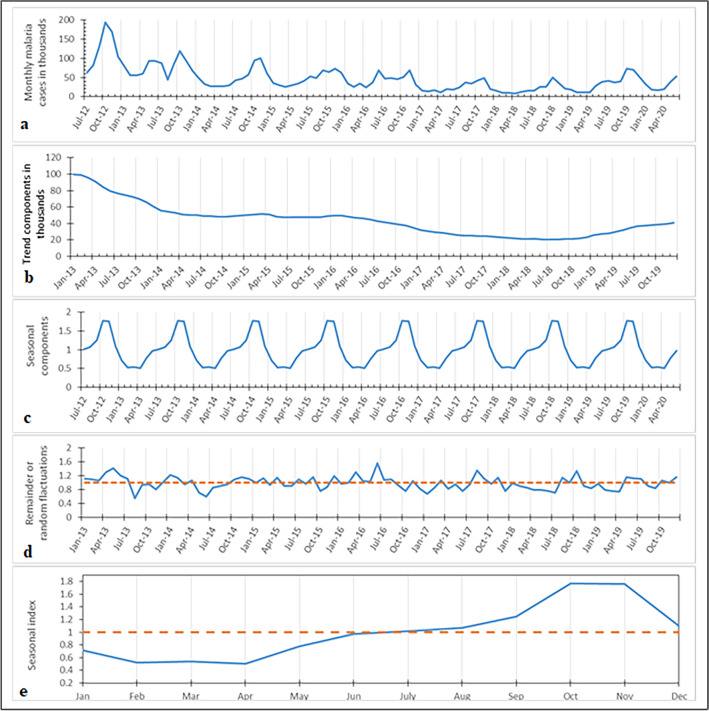
Seasonal distribution of monthly malaria cases between 2012 and 2020, in northwest Ethiopia; (**a**) time series of monthly malaria cases of the region; (**b**) is a long term trend; (**c**) is a seasonal trend decomposed from the time series of malaria cases; (**d**) the residual data after excluding seasonality and long term trend; and (**e**) estimated seasonal index of 12 months.

The time series plot of monthly malaria incidence and its’ components are depicted in a multi-panel plot (Fig. 2). Figure 2a indicates the time series plot of monthly malaria cases in the study region, and it has been decreased between 2012 and 2018 and had a noticeable seasonal variation. The number of cases was in an increased trajectory after the mid of 2018. Figure 2b indicates the long-term trend of monthly malaria cases of the study region, and it has been decreased trend between 2012 and 2018, but it increased from 2019 onwards. Malaria incidence had a seasonal periodicity and was higher from September through December and bottomed down from February to April (Fig. 2c). Figure 1e indicates seasonal index values of months, and the higher seasonal indices were 1.77 and 1.76, which occurred in October and November, respectively. The seasonal indices were smaller from January to June. The temporal clustering analysis also showed that malaria incidence was concentrated in the autumn season, annually ranging from September to November. The range of months of higher temporal clustering was slightly varied across years, especially in 2016, malaria risk was higher in November. A higher temporal clustering of malaria incidence was observed in the study area between July 2012 and December 2013 (LLR = 414,013.41, P < 0.05). During this period, a total of 1,681,173 malaria cases occurred, and the risk of malaria infection was 2.54 times (RR = 2.54, P < 0.05) as compared to the other periods (Table 2).

**Table 2:** Temporal clustering of malaria incidence in the Amhara regional state, Ethiopia, 2012–2020.

Year	Cluster time frame	Observed	Expected cases	RR	LLR	P
2012	2012/9 to 2012/10	324,450	246,887.89	1.56	17,459.67	0.00
2013	2013/9 to 2013/11	298,751	233,474.09	1.41	11,506.08	0.00
2014	2014/10 to 2014/11	194,094	99,800.01	2.40	44,376.23	0.00
2015	2015/9 to 2015/11	207,268	142,453.64	1.71	18,074.49	0.00
2016	2016/11 to 2016/11	69,295	42,633.03	1.72	7,756.24	0.00
2017	2017/9 to 2017/11	125,011	75,754.13	2.10	19,111.17	0.00
2018	2018/9 to 2018/11	111,695	61,812.40	2.47	23,581.39	0.00
2019	2019/10 to 2019/12	196,090	109,705.08	2.43	40,135.85	0.00
2020	2020/6 to 2020/6	53,441	29,769.07	2.13	9,560.05	0.00
2012–2020	2012/7 to 2013/12	1,681,173	852,520.72	2.54	414,013.41	0.00

*LLR* log-likelihood ratio, *RR* relative risk, *P* p-value.

### Spatial cluster analysis

**Figure 3:**
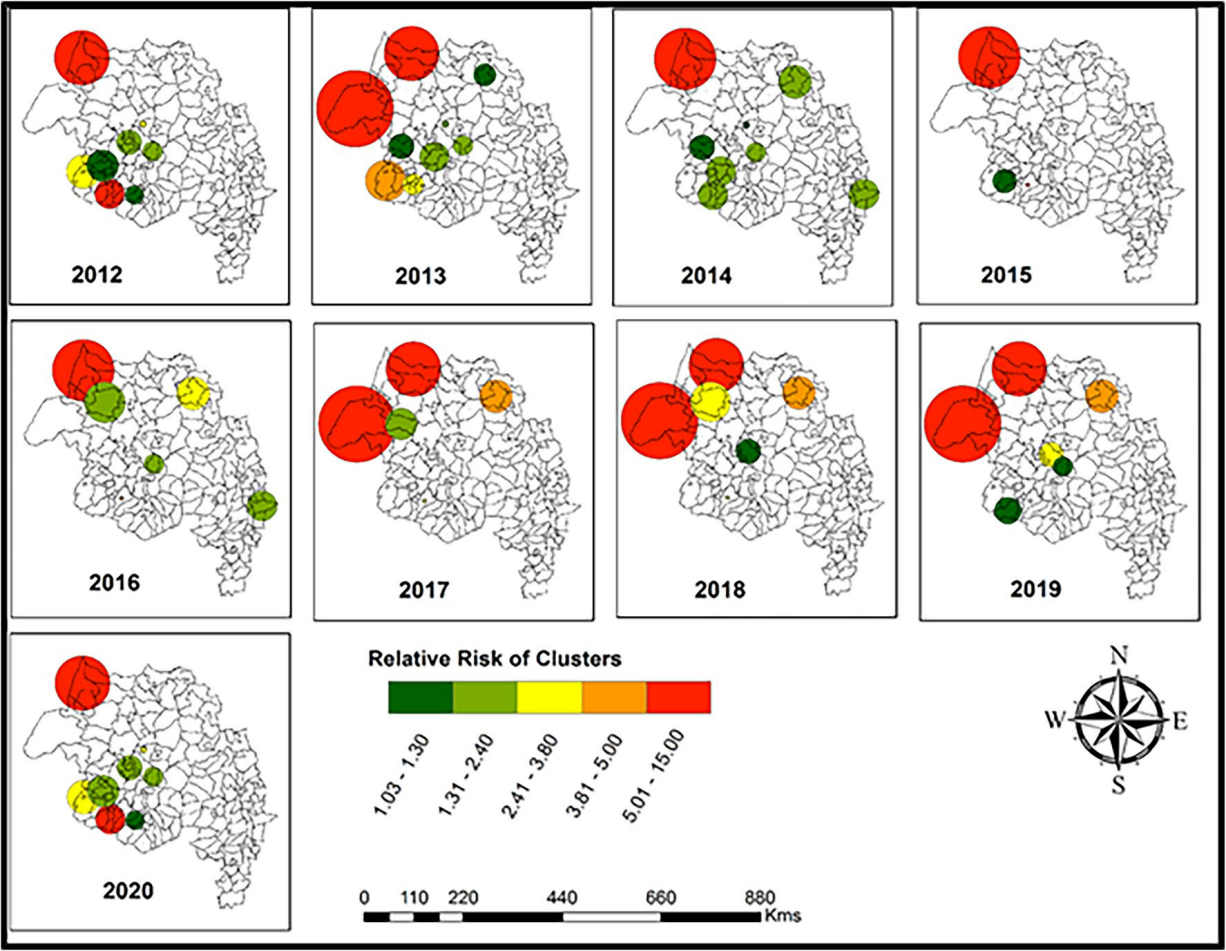
Detected purely spatial clusters using a discrete Poisson model in northwest Ethiopia, 2012–2020.

Purely spatial scan statistics were employed to detect spatial clusters with elevated risks in each year between 2012 and 2020 and presented in Fig. 3. We detected 16 non-overlapping clusters using Gini coefficient methods for each year, and the most likely clusters are summarized in Table S1. The radius of the most likely cluster was ranged from 33.1 to 85.71 km. The most likely reported spatial clusters included 4 to 5 districts in 2012 -2020, with a range of observed malaria cases between 71,558 and 131,918.

The districts included in the most likely purely spatial clusters were consistently homogeneous between 2014 and 2016. Similarly, districts in the most likely clusters are consistently similar in 2013 and 2017–2019. Moreover, districts included in the most likely clusters were consistently similar between 2012 and 2020 (Fig. 3). The annual malaria incidence spatial clustering revealed that the most likely clusters occurred in the northern and northwestern parts of the study region and had higher relative risks than other regions (Fig. 3: shaded in red color).

### Spatiotemporal cluster analysis

**Figure 4:**
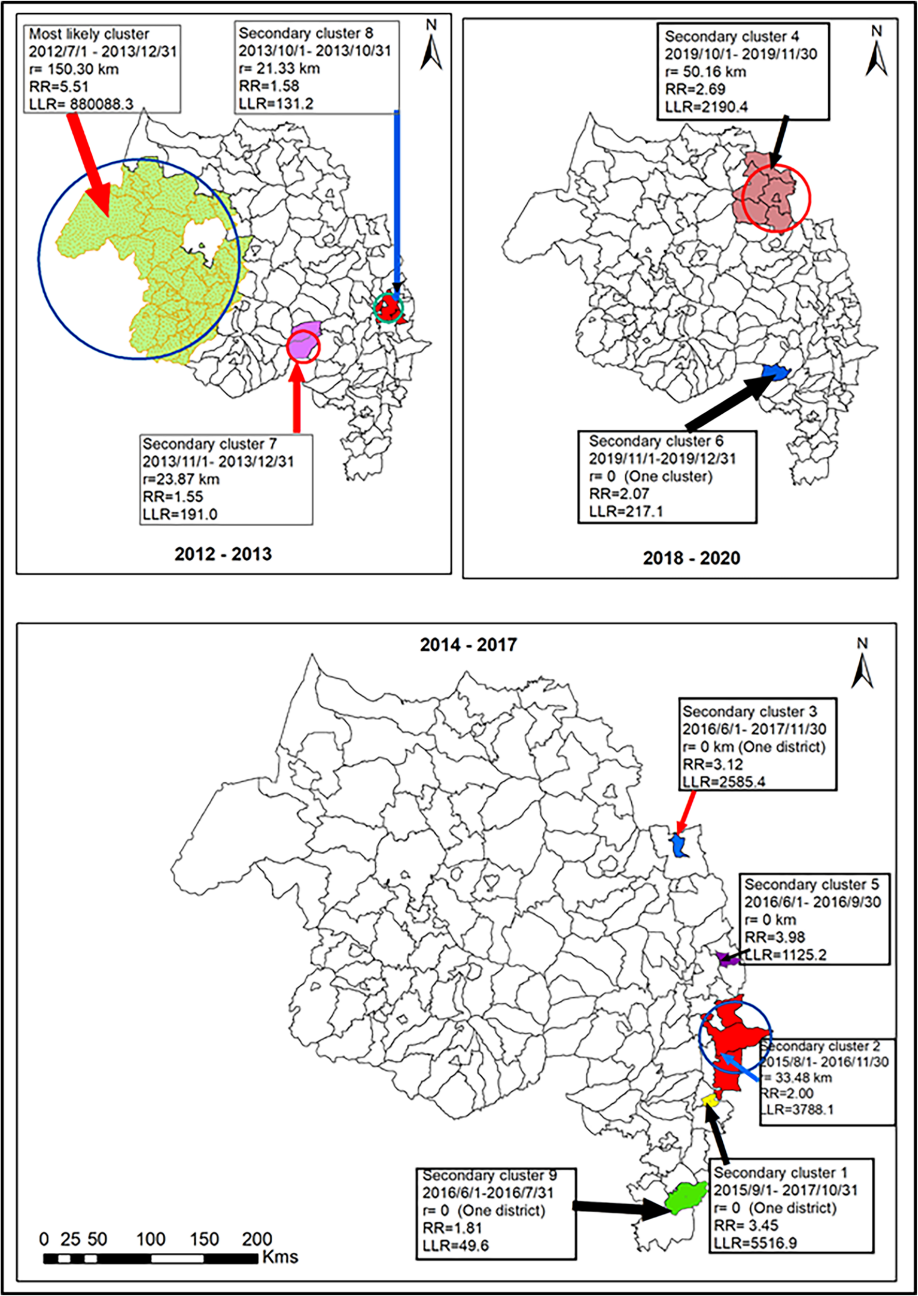
Spatiotemporal clusters of malaria cases at district level without adjusting to the altitude and LLINS covariates in northwest Ethiopia, 2012–2020.

Kulldorff’s retrospective space–time scan statistics using a discrete Poisson model were used to detect spatiotemporal clusters of monthly malaria cases at the district level from July 2012 to June 2020. Table S2 and Fig. 4 present the space–time scanning results of the most likely and the nine secondary statistically significant spatiotemporal clusters of malaria incidence in the study area. The most likely spatiotemporal cluster has included 36 districts; however, less than seven districts were included in each significant secondary cluster. The more likely space–time cluster was located in the west and northwest parts of the region, and a high-risk period was from July 2012 to December 2013 (LLR = 880,088.3, RR = 5.5, P < 0.05). The secondary space–time clusters were located in the northeastern, eastern, southern parts of the region in different periods. The largest secondary spatiotemporal cluster was found in the northeastern parts of the region with six districts, and a high-risk period was between October 2019 and November 2019 (LLR = 2190.4, RR = 2.69, P < 0.05). The risk of malaria was higher in the Oromia special zone of the study region than nearby areas and occurred between August 2016 and November 2016 (LLR = 3788.1, RR = 2.0, P < 0.05).

**Figure 5:**
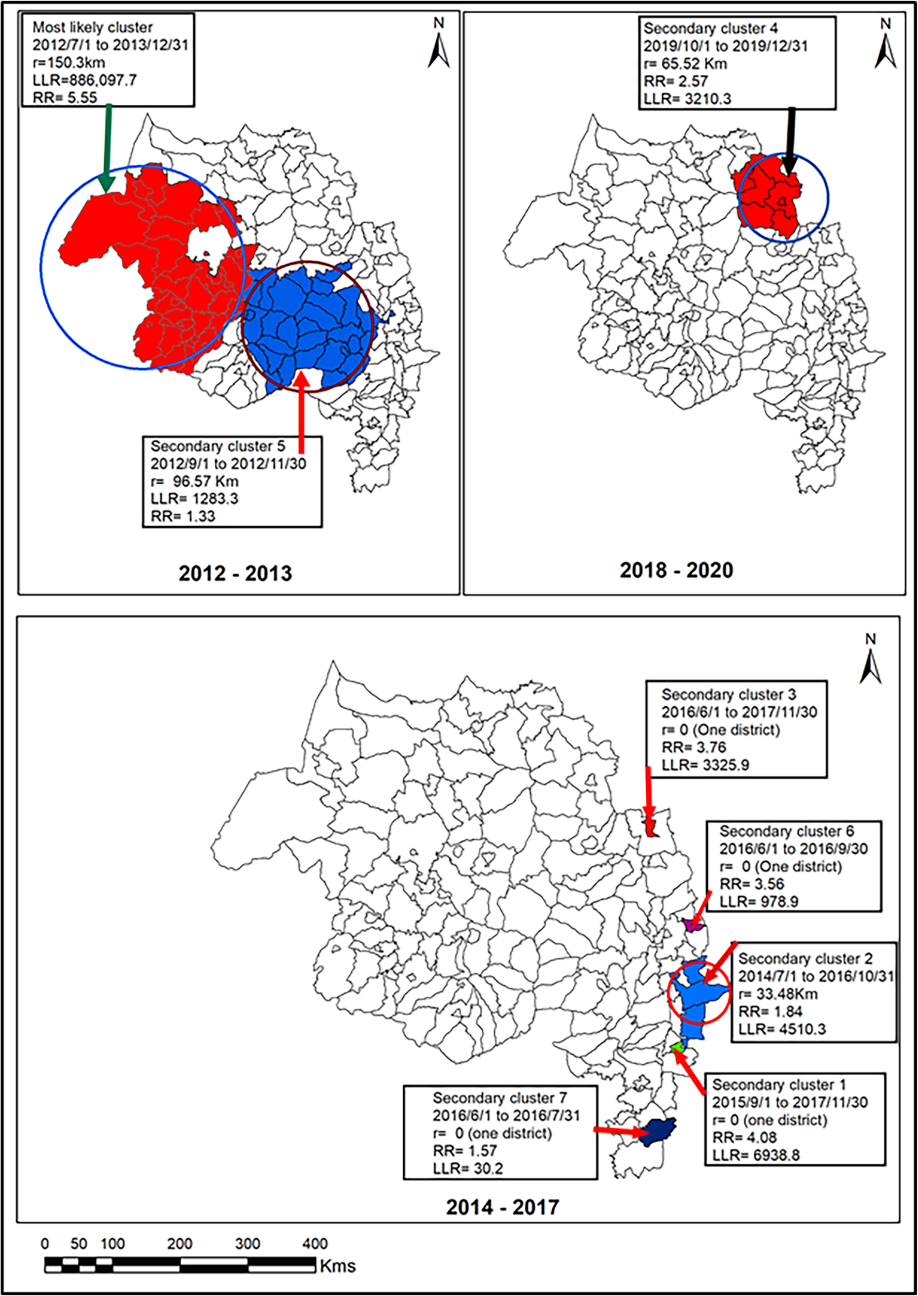
Space–time detection of clusters of malaria cases in the Altitude and LLINS adjustment covariate in northwest Ethiopia, 2012–2020.

Kulldorff’s retrospective space–time scan statistics using a discrete Poisson model under the adjustment of average altitude and LLINS (altitude + LLINS arm) had generated eight clusters and depicted in Fig. 5 and Table S3. We found eight significant spatiotemporal clusters, and the most likely cluster has occurred in the western and northwestern parts of the region, including about 24.45% of malaria cases. The most likely spatiotemporal cluster of altitude + LLIN arm was similar to space–time clusters without adjusting the effects of factors such as average altitude and coverage of LLIN at the household level. The higher malaria risk period of the most likely cluster was between July 2012 and December 2013 (LLR = 886,097.7, RR = 5.55, P < 0.05). We found seven secondary spatiotemporal clusters, and the largest secondary cluster included 28 districts and occurred in the central and southwest parts of the region in September 2012 and November 2012 (LLR = 1283.3, RR = 1.33, P < 0.05). Further, two secondary clusters occurred in the eastern and northeastern parts of the region, specifically in the Wag Himra and Oromia special zone. The higher risk periods of the Wag Himra zone was between October 2019 and December 2019 (LLR = 3210.3, RR = 2.57, P < 0.05); however, it was higher between July 2014 and October 2016 in Oromia special zone (RR = 1.84, LLR = 4510.3, P < 0.05).

We found 28 districts in the secondary cluster 5 cover larger areas than other secondary clusters without adjusting to the altitude + LLIN arm. The higher risk period of secondary clusters with and without adjusting the latitude + LLIN arm occurred in similar time intervals. However, most secondary clusters occurred in an individual district and occured due to malaria epidemics in a higher transmission season between September and December (Fig. 5).

## Discussion

We found a temporal, spatial, and spatiotemporal clustering of malaria incidence in northwest Ethiopia between 2012 and 2020. The temporal clusters using the 20–50% temporal scanning window size of the study period were similar. The detected temporal clusters were identified by setting maximum temporal scanning at the 30% of total study periods, in the ranges of 7–50% of the study population that several authors employed in their works^19,23,35^. During the annual and the whole study period, temporal clusters were detected, and the maximum temporal window sizes for each year were three months. The most likely temporal cluster occurred in northwest Ethiopia's higher malaria transmission season from September to December^4,36^. The malaria epidemic had a seasonal variation and was more prevalent between September and November, but the occurrence months varied from year to year^16,36,37^. Malaria incidence gradually decreased between 2012 and 2018 and increased from 2019 onwards. The most likely temporal clusters of malaria infection were observed in all districts from July 2012 to December 2013.

We constructed purely spatial scan clusters using a discrete Poisson model on yearly total malaria cases at the district level. Determining the optimal maximum spatial cluster size was sensitive to spatial scanning window size, which depends on the percentages of the total population included in the radius of the spatial scan statistics^30,31^. The Gini coefficient was used to identify the optimum maximum spatial cluster sizes, and 16 clusters were detected via clusters having higher Gini coefficients that indicate the presence of higher heterogeneity across districts. Districts were not homogeneous in their malaria transmission, and the most likely annual spatial clusters occurred in the northern and northwestern districts of the study area. Though rigorous malaria interventions have been practiced through the collaborated efforts of government and partners^4,38^, malaria remains the major health burden. Malaria risk was higher, and the most likely clusters occurred in the northern and western parts of the region between 2012 and 2020, similar to research findings that showed clustering of malaria incidence in the western parts of the region^15,37^.

The spatiotemporal cluster analysis provided further evidence for the occurrence of a higher malaria case than the expected arising within a defined place and time. The space–time clusters of malaria risks in the districts of the study area were detected by Kulldorff’s retrospective space–time scan statistics using a discrete Poisson model with and without considering adjustment factors. Malaria incidence has space–time heterogeneity and was clustered into ten groups without adjusting to environmental effects and disparity of LLIN coverage. The most likely space–time cluster covered large areas and occurred in the western and northwestern districts of the study area^1,3^ between 2012 and 2013, which was contradicted to the finding of Bayih et al.^15^ might be due to the inclusion of a smaller number of districts in their study. Though malaria transmission prevention and controlling practices intensified in the country^39^, space–time clustering of malaria incidence was also detected in lower transmission districts of the study area. The secondary clusters were also detected in Oromia and Wag Himra, the two special zones of the Amhara Region, from October 2019 to December 2019 and from July 2015 to December 2016, respectively. Previous studies conducted in the northwest of Ethiopia revealed that all identified clusters were closely related to a specific geographical area and shared similar geographical parameters, such as altitude, weather condition, coverage of LLIN, and indoor residual spraying^13,35^.

Malaria infection is significantly reduced by improving long-lasting insecticide nets and scaling up coverage of indoor residual spraying^38^. Further, malaria epidemiology varies across altitudes of districts, determining the weather condition and having a crucial role in stratifying malaria-prone areas^40^. We identified the space–time clustering of districts with an elevated risk of malaria incidence using altitude + coverage of LLIN usage arm and found eight significant space–time clusters. The most likely spatiotemporal cluster occurred in the western and northwestern parts of the study region and had a higher malaria risk between July 2012 and December 2013 and included nearly one-fourth of all malaria incidence in the study period^10,15,41^. We also found three secondary clusters with more than three districts and occurred in different periods. The altitude + LLIN arm space–time clustering using a discrete Poisson model detected a larger cluster with 28 districts in the western parts of the region between September 2012 and November 2012 in the higher transmission season of the given year. Though malaria incidences were lower in the western and northwestern districts of the region, especially in the Wag Himra and Oromia special zones, malaria is a major public health burden. It had elevated risk compared with neighborhood districts in short to longer periods.

Due to the data availability issue, the space–time clustering of malaria incidence was not considered with adjusting the coverage of indoor residual spraying (IRS) and other environmental covariates. In addition, patients diagnosed in the referral and specialized hospital were excluded from the study due to district-level analysis that patients might come across districts and create fraud clusters at town and city administrations. We used malaria surveillance is archived at the district level. Hence, concerned bodies would consider archiving surveillance data at the institution and lowest administrative level to detect clusters for interventions, specifically in the malaria epidemic areas.

## Conclusions

This study detected the temporal, spatial, and space–time clusters of malaria incidence at the district level in northwest Ethiopia from 2012 to 2020 using Kulldorff’s retrospective space–time scan statistic. The trends of malaria cases have decreased between 2012 and 2018 and increased since 2019. The malaria transmission had seasonal variations and was most prevalent between October and November in the Amhara region. In 2012–2020, the purely temporal clusters occurred between July 2012 and December 2013. Considering the effects of altitudinal variations and coverage of LLIN usage, districts located in the west and northwest parts of the region had a higher malaria burden. Further, secondary space–time clusters were aggregated in the west, northeast, and east of the study regions and occurred in different periods. Considering these space–time variations and factors contributing to the intensification of malaria infection would be useful for prevention and control and ultimately enable achieving the country's malaria elimination goals in 2030.

## Supplementary Information

Supplementary Information.

## Data Availability

The data presented in this study are available on request from the corresponding author. The data are not publicly available due to the data-sharing policy of Amhara Public Health Institute.

## Abbreviations

APHI: Amhara Public Health institute
LLINS: Long-lasting insecticide nets
IRS: Indoor residual spraying
RR: Relative risk
LLR: Log-likelihood ratio
P: P-value
WHO: World Health Organization

## References

[1] Alemu K, Worku A, Berhane Y (2013). Malaria infection has spatial, temporal, and spatiotemporal heterogeneity in unstable malaria transmission areas in northwest Ethiopia. PLoS ONE.

[2] Ayele DG, Zewotir TT, Mwambi HG (2012). Prevalence and risk factors of malaria in Ethiopia. Malar. J..

[3] Midekisa A, Beyene B, Mihretie A, Bayabil E, Wimberly MC (2015). Seasonal associations of climatic drivers and malaria in the highlands of Ethiopia. Parasit. Vectors.

[4] FMOH. NATIONAL MALARIA GUIDELINES Third Edition. Ministry of Health of Federal Democratic Republic of Ethiopia Ababa, Ethiopia (2012).

[5] IRS PMIA. Project Indoor Residual Spraying (IRS) Task Order Six (2016).

[6] Taye G, Kaba M, Woyessa A, Deressa W, Simane B, Kumie A (2015). Modeling effect of climate variability on malaria in Ethiopia. Ethiop J. Heal. Dev..

[7] Bousema T, Griffin JT, Sauerwein RW, Smith DL, Churcher TS, Takken W (2012). Hitting hotspots: Spatial targeting of malaria for control and elimination. PLoS Med..

[8] Alelign A, Tekeste Z, Petros B (2018). Prevalence of malaria in Woreta town, Amhara region, Northwest Ethiopia over eight years. BMC Public Health.

[9] Yalew WG, Pal S, Bansil P, Dabbs R, Tetteh K, Guinovart C (2017). Current and cumulative malaria infections in a setting embarking on elimination: Amhara, Ethiopia. Malar. J..

[10] Taddese AA, Baraki AG, Gelaye KA (2019). Spatial modeling, prediction, and seasonal variation of malaria in northwest Ethiopia. BMC Res. Notes.

[11] Zemene E, Belay DB, Tiruneh A, Lee M-C, Yewhalaw D, Yan G (2021). Malaria vector dynamics and utilization of insecticide-treated nets in low-transmission setting in Southwest Ethiopia: Implications for residual transmission. BMC Infect. Dis..

[12] Coleman M, Coleman M, Mabuza AM, Kok G, Coetzee M, Durrheim DN (2009). Using the SaTScan method to detect local malaria clusters for guiding malaria control programmes. Malar. J..

[13] Id TS, Loha E, Deressa W, Gari T, Lindtj B (2019). Spatiotemporal clustering of malaria in southern-central Ethiopia: A community-based cohort study.

[14] Kibret S, Wilson GG, Ryder D, Tekie H, Petros B (2017). The influence of dams on malaria transmission in sub-Saharan Africa. EcoHealth.

[15] Bayih, E.T., Gelaye, K.A., Zeleke, A.D., Damtew, A.S., Asmare, B.A., & Demil, Y.A., et al. Spatial, temporal, and spatiotemporal variation of malaria incidence and risk factors in West Gojjam zone from 1 July 2013–30 June 2018, Northwest Ethiopia, 2019 (2020).

[16] Taye G, Kaba M, Woyessa A, Deressa W, Simane B, Kumie A (2015). Modeling effect of climate variability on malaria in Ethiopia. Ethiop J. Heal. Dev..

[17] Lankir D, Solomon S, Gize A (2020). A five-year trend analysis of malaria surveillance data in selected zones of Amhara region Northwest Ethiopia. BMC Public Health.

[18] Taffese HS, Hemming-Schroeder E, Koepfli C, Tesfaye G, Lee MC, Kazura J (2001). Malaria epidemiology and interventions in Ethiopia from 2001 to 2016. Infect. Dis. Poverty.

[19] Seyoum D, Yewhalaw D, Duchateau L, Brandt P, Rosas-Aguirre A, Speybroeck N (2017). Household level spatio-temporal analysis of Plasmodium falciparum and Plasmodium vivax malaria in Ethiopia. Parasit. Vectors.

[20] CSA. Population and Housing Census 2007 Report. Cent Stat Agency, Addis Ababa (2007).

[21] ESRI. ArcGIS Desktop: Release 10.3. Redlands, CA Environ Syst Res Inst (2011).

[22] CSA. Population projection of ethiopia for all regions at Wereda level from 2014 to 2017. *J. Ethnobiol. Ethnomed.* (2013).

[23] Wu X, Hu S, Kwaku AB, Li Q, Luo K, Zhou Y (2017). Spatio-temporal clustering analysis and its determinants of hand, foot and mouth disease in Hunan, China, 2009–2015. BMC Infect. Dis..

[24] Lee CF (2021). Time-series analysis: Components, models, and forecasting.

[25] Zhang Q, Lai S, Zheng C, Zhang H, Zhou S, Hu W (2014). The epidemiology of Plasmodium vivax and Plasmodium falciparum malaria in China, 2004–2012: from intensified control to elimination. Malar. J..

[26] Kulldorff M, Huang L, Pickle L, Duczmal L (2006). An elliptic spatial scan statistic. Stat. Med..

[27] Kulldorff M, Feuer EJ, Miller BA, Freedma LS (1997). Breast cancer clusters in the northeast United States: A geographic analysis. Am. J. Epidemiol..

[28] Kulldorff M, Heffernan R, Hartman J, Assunçao R, Mostashari F (2005). A space–time permutation scan statistic for disease outbreak detection. PLoS Med.

[29] Kulldorff, M. Communications in statistics—Theory and methods A spatial scan statistic 37–41 (2007).

[30] Ma Y, Yin F, Zhang T, Zhou XA, Li X (2016). Selection of the maximum spatial cluster size of the spatial scan statistic by using the maximum clustering set-proportion statistic. PLoS ONE.

[31] Han J, Zhu L, Kulldorff M, Hostovich S, Stinchcomb DG, Tatalovich Z (2016). Using Gini coefficient to determining optimal cluster reporting sizes for spatial scan statistics. Int. J. Health Geogr..

[32] Tango T, Takahashi K (2005). A flexibly shaped spatial scan statistic for detecting clusters. Int. J. Health Geogr..

[33] Tango T, Takahashi K (2012). A flexible spatial scan statistic with a restricted likelihood ratio for detecting disease clusters. Stat. Med..

[34] Solomon T, Loha E, Deressa W, Gari T, Overgaard HJ, Lindtjørn B (2019). Low use of long-lasting insecticidal nets for malaria prevention in south-central Ethiopia: A community-based cohort study. PLoS ONE.

[35] Rao, H., Shi, X., & Zhang, X. Using the Kulldorff ’ s scan statistical analysis to detect spatio-temporal clusters of tuberculosis in Qinghai Province, China. 1–11. 10.1186/s12879-017-2643-y (2017). 10.1186/s12879-017-2643-y PMC5563899 28826399

[36] Yewhalaw D, Getachew Y, Tushune K, Kassahun W, Duchateau L, Speybroeck N (2013). The effect of dams and seasons on malaria incidence and anopheles abundance in Ethiopia. BMC Infect. Dis..

[37] Taddese AA, Baraki AG, Gelaye KA (2019). Spatial modeling, prediction and seasonal variation of malaria in northwest Ethiopia. BMC Res. Notes.

[38] Loha E, Deressa W, Gari T, Balkew M, Kenea O, Solomon T (2019). Long-lasting insecticidal nets and indoor residual spraying may not be sufficient to eliminate malaria in a low malaria incidence area: Results from a cluster randomized controlled trial in Ethiopia. Malar. J..

[39] FMOH. National Malaria Guidelines, Fourth edition. Natl Malar Giuideline, 3ed edn, pp. 1–108 (2017).

[40] Pascual M, Bouma MJ (2009). Do rising temperatures matter?. Ecology.

[41] Alemu K, Worku A, Berhane Y, Kumie A (2014). Spatiotemporal clusters of malaria cases at village level, northwest Ethiopia. Malar. J..

