# Supplementary material for: Detection of temporal, spatial and spatiotemporal clustering of malaria incidence in northwest Ethiopia, 2012–2020
Source: Sci Rep. 2022 Mar 7;12:3635. doi: 10.1038/s41598-022-07713-3 (PMC8901673; doi:10.1038/s41598-022-07713-3)
Supplement: Supplementary file 1 — Supplementary Information. [file 41598_2022_7713_MOESM1_ESM.pdf]

Detection of temporal, spatial and spatiotemporal clustering of malaria incidence in northwest Ethiopia, 2012 – 2020

Teshager Zerihun Nigussie[1*], Temesgen T. Zewotir[2] and Essey Kebede Muluneh[3]

[1] Department of Statistics, College of Science, Bahir Dar University, Bahir Dar- Ethiopia

[2] School of Mathematics, Statistics and Computer Science, College of Agriculture Engineering and Science, University of KwaZulu-Natal, Durban, South Africa

[3]Department of public health, College of Medicine and Health Sciences, Bahir Dar University, Bahir Dar- Ethiopia

Table S1: Spatial clustering of annual malaria cases using purely spatial scan statistics in northwest Ethiopia, 2012 - 2020.

Year	*Coordinates/radius (km)*	*N*	*Observed*	*Expected*	*RR*	*LLR*	*P*
2012	(10.516000 N, 37.045000 E) / 33.10 km	5	124,704	20,103.2	7.25	130,933.6	0.00
2013	(12.219000 N, 35.863000 E) / 85.71 km	4	131,918	24,512.8	6.10	121,199.5	0.00
2014	(13.248000 N, 36.452000 E) / 68.95 km	5	109,059	12,099.7	10.80	151,348.3	0.00
2015	(13.248000 N, 36.452000 E) / 68.95 km	5	120,795	11,531.1	13.02	185,921.7	0.00
2016	(13.248000 N, 36.452000 E) / 68.95 km	5	92,641	10,595.4	10.42	125,820.7	0.00
2017	(12.219000 N, 35.863000 E) / 85.71 km	4	80,271	7,934.2	13.40	123,094.0	0.00
2018	(12.219000 N, 35.863000 E) / 85.71 km	4	71,558	6,531.4	15.00	115,946.8	0.00
2019	(12.219000 N, 35.863000 E) / 85.71 km	4	89,646	11,402.4	9.64	114,323.5	0.00
2020	(10.516000 N, 37.045000 E) / 33.10 km	5	124,704	20,103.2	7.25	130,933.6	0.00

N: Number of districts; RR: Relative risk; LLR: Log-likelihood ratio; P: P-value

Table S2: Spatiotemporal clusters of malaria cases in northwest Ethiopia, 2012 - 2020.

Type	*N*	*Time frame*	*Coordinates/radius (kms)*	*Population*	*Observed*	*Expected*	*RR*	*LLR*	*P*
MC	36	2012/7/1 to 2013/12/31	(11.717 N, 36.411 E) / 150.30 km	*1,339,544*	*1,109,343*	*251,680.8*	*5.51*	*880,088.3*	*0.00*
SC1	1	2015/9/1 to 2017/10/31	(9.996 N, 39.893 E) / 0 km	*11,215*	*10,464*	3,039.8	3.45	5,516.9	0.00
SC2	4	2015/8/1 to 2016/11/30	(10.536 N, 40.11 E) / 33.48 km	*58,533*	*19,553*	9,775.6	2.00	3,788.1	0.00
SC3	1	2016/6/1 to 2017/11/30	(12.161 N, 39.624 E) / 0 km	*9,639*	*5,639*	1,807.7	3.12	2,585.4	0.00
SC4	6	2019/10/1 to 2019/11/30	(12.616 N, 39.039 E) / 50.16 km	*108,014*	*6,063*	2,254.9	2.69	2,190.4	0.00
SC5	1	2016/6/1 to 2016/9/30	(11.194 N, 40.041 E) / 0 km	*10,708*	*1,779*	447.1	3.98	1,125.2	0.00
SC6	**1**	2019/11/1 to 2019/12/31	(10.244 N, 39.008 E) / 0 km	*23,669*	*1,025*	494.1	2.07	217.1	0.00
SC7	2	2013/11/1 to 2013/12/31	(10.539 N, 38.675 E) / 23.87 km	*71,198*	*2,301*	1,486.3	1.55	191.0	0.00
SC8	3	2013/10/1 to 2013/10/31	(11.067 N, 39.842 E) / 21.33 km	*85,946*	*1,443*	911.8	1.58	131.2	0.00
SC9	1	2016/6/1 to 2016/7/31	(9.186 N, 39.685 E) / 0 km	*8,956*	*339*	187.0	1.81	49.6	0.00

MC: the most likely cluster; SC: Secondary Cluster; N: number of districts; RR: Relative risk, LLR: Log-likelihood ratio; P: P-value

Table S3: Spatiotemporal clustering of reported malaria cases using altitude and LLIN arms (adjustments) in northwest Ethiopia between 2012 and 2020.

Type	*N*	*Time frame*	*Coordinates/radius (km)*	*Population*	*Observed*	*Expected*	*RR*	*LLR*	*P*
MC	36	2012/7/1 to 2013/12/31	(11.717 N, 36.411 E)/ 150.3km	*1,339,544*	*1,109,343*	*250,021.9*	*5.55*	*886,097.7*	*0.00*
SC1	1	2015/9/1 to 2017/11/30	(9.996 N, 39.893 E)/ 0km	*11,215*	*10,678*	2,624.3	4.08	6,938.8	0.00
SC2	4	2014/7/1 to 2016/10/31	(10.536 N, 40.11 E)/ 33.48km	*58,533*	*29,617*	16,154.0	1.84	4,510.3	0.00
SC3	1	2016/6/1 to 2017/11/30	(12.161 N, 39.624 E)/ 0km	*9,639*	*5,639*	1,501.6	3.76	3,325.9	0.00
SC4	7	2019/10/1 to 2019/12/31	(12.667 N, 39.063 E)/ 65.52km	*114,788*	*9,685*	3,780.4	2.57	3,210.3	0.00
SC5	28	2012/9/1 to 2012/11/30	(10.937 N, 38.645 E)/ 96.57km	*936,764*	*35,495*	26,811.6	1.33	1,283.3	0.00
SC6	1	2016/6/1 to 2016/9/30	(11.94 N, 40.041 E)/ 0 km	*10,708*	*1,779*	500.1	3.56	978.9	0.00
SC7	1	2016/6/1 to 2016/7/31	(9.186 N, 39.685 E)/ 0km	*8959*	*339*	215.3	1.57	30.2	0.00

MC: the most likely cluster; SC: Secondary Cluster; N: number of districts; RR: Relative risk; LLR: Log-likelihood ratio
